# Supplementary material for: Turning inward in difficulties: R&D human resource slack, technological diversification, and independent innovation
Source: PLoS One. 2024 Jun 13;19(6):e0298453. doi: 10.1371/journal.pone.0298453 (PMC11175520; doi:10.1371/journal.pone.0298453)
Supplement: Attachment — Submitted filename: Plos review 3.docx [file pone.0298453.s002.docx]

Thank you for the opportunity to review your paper.

The research paper discusses how internal human resource R & D slack can be used to develop independent innovation capabilities. It also explores the role of top management team’s functional heterogeneity and the organization’s technological diversification. The study is relevant to manufacturing industry facing an uncertain environment. The study is interesting. However, some areas of improvement are given below:

Introduction

1. It is unclear whether the slack literature refers to human resource slack. If it is not, past literature on human resource slack needs to be included, specifically in R & D area.
2. Elaboration is needed on how is independent innovation different from the regular innovation activities and how does an enterprise manage it differently
3. The research gap needs further clarification

Theoretical basis and research hypotheses

4. Independent innovation needs to be defined in contrast to standard innovation
5. Existing literature on the implication of human resource slack on innovation is missing.
6. It is not clear why human resource R & D slack will promote independent innovation specifically, and not innovation in general.
7. Relevant literature on technological diversification and TMT functional heterogeneity needs to be added. Also, the respective mediation and moderation hypotheses needs to have stronger arguments

Research design

8. The argument on choosing patents as a measure of independent innovation needs to be more convincing. Standard innovation may also use the same measure.
9. It needs to be clarified whether you are doing correlational or causal analysis. The hypotheses need to be modified accordingly
10. Please include how you have accounted for long innovation cycles and time taken for patenting. It is not necessary that the patents are obtained in the same year in which the R & D slack exists. Also, the human resources may not come with an innovation within a year. Same holds for the intervening and control variables
11. You have mentioned that there are restrictions for making the data fully available. Please provide reasons for the same.

Conclusion and Discussion

12. The theoretical implications need to indicate how the results are contributing to existing literature related to the variables and theories. Currently, it seems to be an extension of the conclusion section. The theoretical implications are not apparent.
13. The conclusion should follow the discussion as a separate section
